# Simultaneous vehicle and lane detection via MobileNetV3 in car following scene

**DOI:** 10.1371/journal.pone.0264551

**Published:** 2022-03-04

**Authors:** Tianmin Deng, Yongjun Wu

**Affiliations:** 1 School of Automation, Chongqing University, Chongqing, China; 2 School of Traffic & Transportation, Chongqing Jiaotong University, Chongqing, China; Torrens University Australia, AUSTRALIA

## Abstract

Aiming at vehicle and lane detections on road scene, this paper proposes a vehicle and lane line joint detection method suitable for car following scenes. This method uses the codec structure and multi-task ideas, shares the feature extraction network and feature enhancement and fusion module. Both ASPP (Atrous Spatial Pyramid Pooling) and FPN (Feature Pyramid Networks) are employed to improve the feature extraction ability and real-time of MobileNetV3, the attention mechanism CBAM (Convolutional Block Attention Module) is introduced into YOLOv4, an asymmetric network architecture of "more encoding-less decoding" is designed for semantic pixel-wise segmentation network. The proposed model employed improved MobileNetV3 as feature ex-traction block, and the YOLOv4-CBAM and Asymmetric SegNet as branches to detect vehicles and lane lines, respectively. The model is trained and tested on the BDD100K data set, and is also tested on the KITTI data set and Chongqing road images, and focuses on the detection effect in the car following scene. The experimental results show that the proposed model surpasses the YOLOv4 by a large margin of +1.1 AP50, +0.9 Recall, +0.7 F1 and +0.3 Precision, and surpasses the SegNet by a large margin of +1.2 IoU on BDD100k. At the same time, the detection speed is 1.7 times and 3.2 times of YOLOv4 and SegNet, respectively. It fully proves the feasibility and effectiveness of the improved method.

## 1. Introduction

Accurate road environment perception and understanding are crucial for the realization of autonomous driving, which provide the decision system to operate the vehicle with information including: locations, lanes, obstacles, drivable area, and so on. Currently, the environment perception technologies of automatic driving mainly include laser radar, millimeter wave radar, video, image detection and other technologies [1]. Among them, the laser radar has high detection accuracy and can capture the three-dimensional coordinates of target, distance length, azimuth angle, laser reflection intensity, and laser coding parameters at any time. However, the cost of laser radar is very high, though it has been used to detect lane lines [2, 3] and vehicle [4]. Cameras and image sensor are widely used in environment perception because of its low-cost and similarity to that of human visual system. Image is more suitable for human understanding, and it is convenient for human to label the image.

Recently, object detections based on image data in road scenes are paid considerable attention for autonomous driving. For the vehicle detection, the R-CNN [5, 6] and YOLO [7] series networks are usually employed. The SCNN [8] and SAD-ENet [9] networks are used for lane line detection. However, these methods usually handle vehicle or lane line detection tasks separately. Processing the detection tasks one after another takes longer time and more memory though the excellent performance these methods achieve, which would cause lower real-time for autonomous driving.

Multi-Task Learning uses same backbone to extract feature and multi heads to deal with multitasks [10]. It could be more efficient for multi detection tasks for autonomous driving. Therefore, in this paper, we attempt to design a simple and efficient network to detect vehicle and lane line at the same time. The remainder of this paper is organized as follows. some related works of vehicle detection and lane detection reviewed in Section 2. Section 3 offers the proposed vehicle and lane detection network, and the experiments and results of the proposed method for vehicle detection and lane detection are shown in Section 4. Section 5 presents our conclusion and future work.

## 2. Related works

### 2.1. Vehicle detection

Vehicle detection is an important for intelligent transportation systems and autonomous driving technology. It has high practical significance and application prospects for sensing road conditions, coordinating traffic scheduling, alleviating traffic jams and avoiding driving risks. Vision-based vehicle detection methods belong to the category of target detection in the field of computer vision, and mainly use various image processing algorithms to obtain the location and category information of the target vehicle from the captured road images. Based on the research status at home and abroad, vehicle detection technology can be roughly divided into three categories: based on artificial design features, model based and based on convolutional neural network. Because model-based vehicle detection methods are only effective for some specific vehicle targets, they have greater limitations and have low practical application value. Therefore, the commonly used detection methods have been mainly based on two types of methods, artificial design features and convolutional neural networks.

In the feature design, the LBP (Local Binary Pattern) operator is proposed to extract the local texture features of the image in [11], which has the advantage of gray invariance [12]. Believes that the appearance of the region of interest can be represented by its local feature distribution, and designed a new texture feature GoLBP (Grid of LBP) based on LBP for real-time vehicle detection [13]. Proposed the HOG (Histogram of Oriented Gradient) operator, which obtained the directional gradient histogram to characterize the target feature by counting the image gradient information of the local area, and used the contrast normalization technology to improve the impact of feature on the illumination changes. adaptability. On the classifier, SVM [14] (Support Vector Machines) and Adaboost [15] models in machine learning are often used to train and classify the extracted image features [16]. Used Harr-like feature cascade Adaboost classifier method for vehicle detection, and achieved multi-target vehicle tracking through an adaptive Kalman filter.

The method based on convolutional neural network takes the vehicle as the identification target, and through the production of vehicle data sets, improvements and training are carried out on the existing target detection network framework to realize the function of vehicle detection [17]. Proposed a vehicle detection method based on the YOLOv2 framework. By optimizing model parameters, expanding the grid size, and improving the number and size of anchor points, real-time vehicle detection with high accuracy is achieved. Zhang Fukai et al. proposed a method that can detect vehicle types in urban traffic monitoring in real time. This method is called a single-stage deep neural network (DF-YOLOv3) [18]. Proposed a Faster-RCNN network SAR image vehicle target detection method based on improved RPN, which solves the problem that the candidate region generation network (RPN) module in the traditional Faster-RCNN method does not sufficiently extract target features during target detection.

### 2.2. Lane detection

Lane line detection is one of the important technologies for assisted driving and unmanned driving in the road environment perception module. It can provide the necessary decision-making basis for autonomous navigation, merging assistance and lane departure warning and other intelligent driving technologies. At present, lane line detection methods are mainly divided into traditional lane line detection methods and detection methods based on deep learning.

For the traditional lane line detection method, the focus is on the design of the lane line feature extraction method [19]. Because the artificially designed feature extraction method is fixed, it is difficult to flexibly apply to other practical situations, and the use environment is limited by the algorithm. Therefore, a series of improved algorithms came into being. Though variable spacing scanning and double ROI is proposed for lane line detection, it only extracts features from the left and right lane line regions, which improves the real-time detection.

For the deep learning method, it is mainly to establish an artificial neural network model with multiple hidden layers, and extract the more essential features of the target from a large amount of data through network training to ensure the improvement of classification accuracy. A fine database of lane lines is established in [20]. The convolutional neural networks is employed to extract and classify lane line features in [21]. [22] proposed a spatial convolutional neural network, which is suitable for detecting slender continuous shapes. An instance segmentation method to detect lane lines is proposed in [23]. The SegNet [24] is employed to making the lane. Although the convolutional neural network has high accuracy for target extraction, it is still difficult to extract linear regions such as lane lines, and the real-time performance is poor.

### 2.3. Our work

Through there are many works which focus on the vehicle and lane line detection and obtain good performance of accuracy, most of them consider using single model to deal with single task and the real-time performance is poor. In order to balance the efficient and accuracy of vehicle detection and lane line recognition, a lightweight multi-task joint detection network is designed in this paper.

Compared with other lightweight network models (such as: VGG, ResNet18, SqueezeNet [25], ShuffleNetv1-v2 [26, 27] and MobileNetV1-V3 [28–30], etc.), the MobileNetV3 network holds smaller size, less calculation and higher accuracy, and has great advantages in lightweight neural networks. Therefore, this paper chooses this network as the feature extraction network of the vehicle and lane line detection algorithm. In the tasks of target detection and semantic segmentation, the different size of the target increases the difficulty of segmentation and detection. Currently, there are two types of mainstream methods for solving different target sizes. The first type is to use different sizes of convolutions when extracting features. The typical ones are spatial convolutional pooling pyramid network (SPP-Net) [31] and void space. Atrous Spatial Pyramid Pooling (ASPP) [32], the second category is to use feature maps of different sizes for task operations, the most classic feature map is pyramid network (FPN) [33]. Besides, the attention mechanism indicates good improvement of performance, and the Convolutional block attention module [34] could be introduced to YOLOv4 for vehicle detection.

In proposed method, the two tasks are integrated into a deep learning network, and MobileNetV3 is recommended as feature extraction block, and the YOLOv4 and SegNet are employed as branches to detect vehicles and lane lines, respectively. Further, Both ASPP and FPN are employed to improve the feature extraction ability and real-time of MobileNetV3, the attention mechanism CBAM is introduced into YOLOv4, an asymmetric network architecture of "more encoding-less decoding" is designed for semantic pixel-wise segmentation network.

## 3. Proposed MobileNetV3-based vehicle and lane joint detection method

### 3.1. Proposed network architecture

Considering that the network for vehicle detection and lane line segmentation tasks is composed of three parts: backbone network, feature processing and detection task processing. Among the first two parts, the backbone network and feature processing are the commonalities of the two, and the main difference lies in the detection task processing part. This paper is mainly applicable to the vehicle and lane line detection in the car following scene, so it is necessary to detect the most typical targets in the car following scene, that is, the vehicle and the lane line. A model that needs to detect two different types of targets at the same time shows that this model needs to have two task branches: the detection of vehicles and lane lines, and the framework of multi-task combination comes from this. And the framework is also reflected in current face recognition system, which not only detects the position of the face, but also detects the various expressions of the person. Or in the process of instance segmentation, the network of instance segmentation must not only detect the bounding box of the target but also detect its classification and mask, so there are three tasks to be processed in the network of instance segmentation.

**Fig 1:**
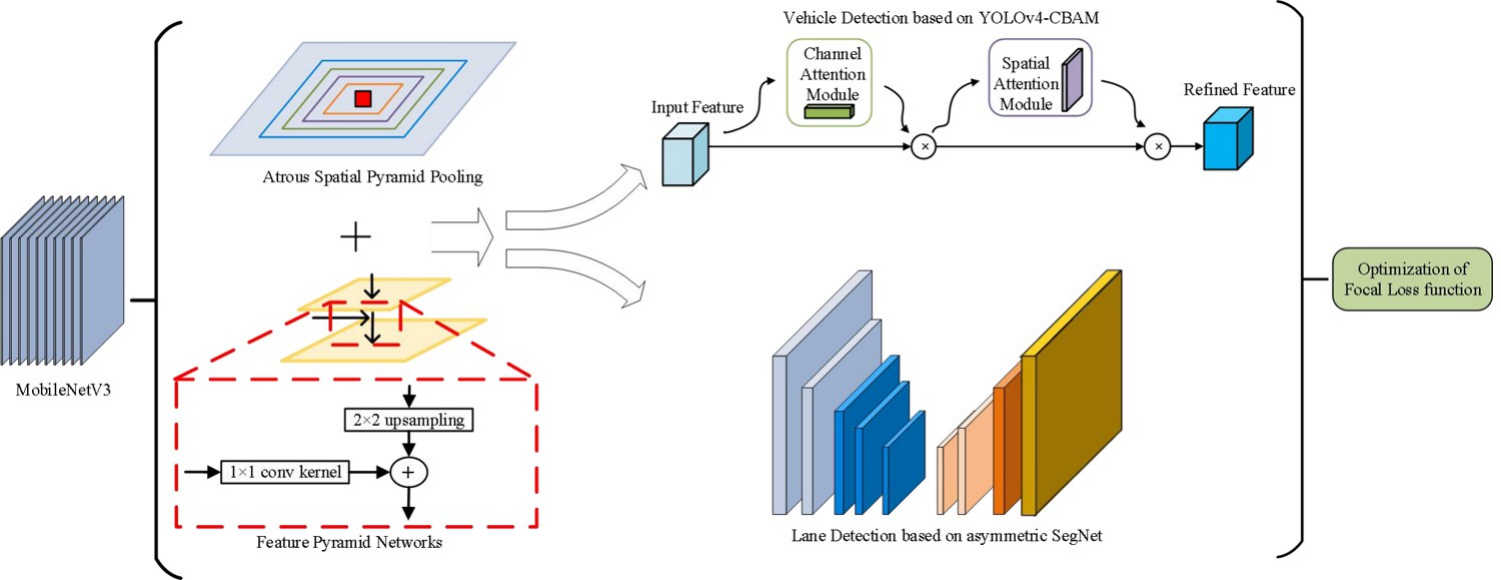
Architecture of proposed vehicle and lane detection.

The multi-task learning method of real-time target detection and semantic segmentation based on lightweight network mainly includes four parts, which are feature extraction part, multi-scale receptive field part, target detection and semantic segmentation part. As a result, the vehicle and lane line multi-task detection model suitable for the car following scene can be constructed in this paper. The flow chart of the connection of each part of the model is shown in Fig 1. As can be seen from the figure, this article will use the Mobilenet V3 network to extract features and send them to the detection part of the upper asymmetric SegNet network to complete the segmentation of the road travelable area and the selectable travel area, thereby detecting lane lines. At the same time, the features are sent to the detection part of the lower YOLOv4-CBAM network to complete the vehicle detection. Next, the receptive field of the feature map is added through the multi-scale receptive field, and the convolution of different scales is used to solve the multi-scale problem. The final loss function will pass the loss function of the detection module of the asymmetric SegNet network and the loss function of the YOLOv4-CBAM network. The loss function of the detection module is weighted and summed according to a certain weight coefficient, so that the overall model is optimized.

### 3.2. Improved MobileNetV3 block for feature extraction

#### 3.2.1. Multi-scale receptive field module

ASPP mainly has two functions: expanding the receptive field and capturing multi-scale contextual information. The first is to expand the receptive field function. In the classic deep convolutional network, in addition to the convolutional layer and the activation layer, a pooling layer is also introduced. Generally, after the pooling layer, the size of the feature map will become smaller, and the amount of calculation will be reduced. Field expansion plays an important role, but the reduction in spatial resolution caused by the reduction of the feature map size will cause the loss of spatial semantic information, which will have a great impact on the vehicle detection task branch and the lane line detection task branch. The hole convolution can expand the receptive field without reducing the spatial resolution, and can better detect and segment large targets. At the same time, the network retains as much spatial information as possible to improve the positioning accuracy of the target position. Very useful in semantic segmentation.

**Fig 2:**
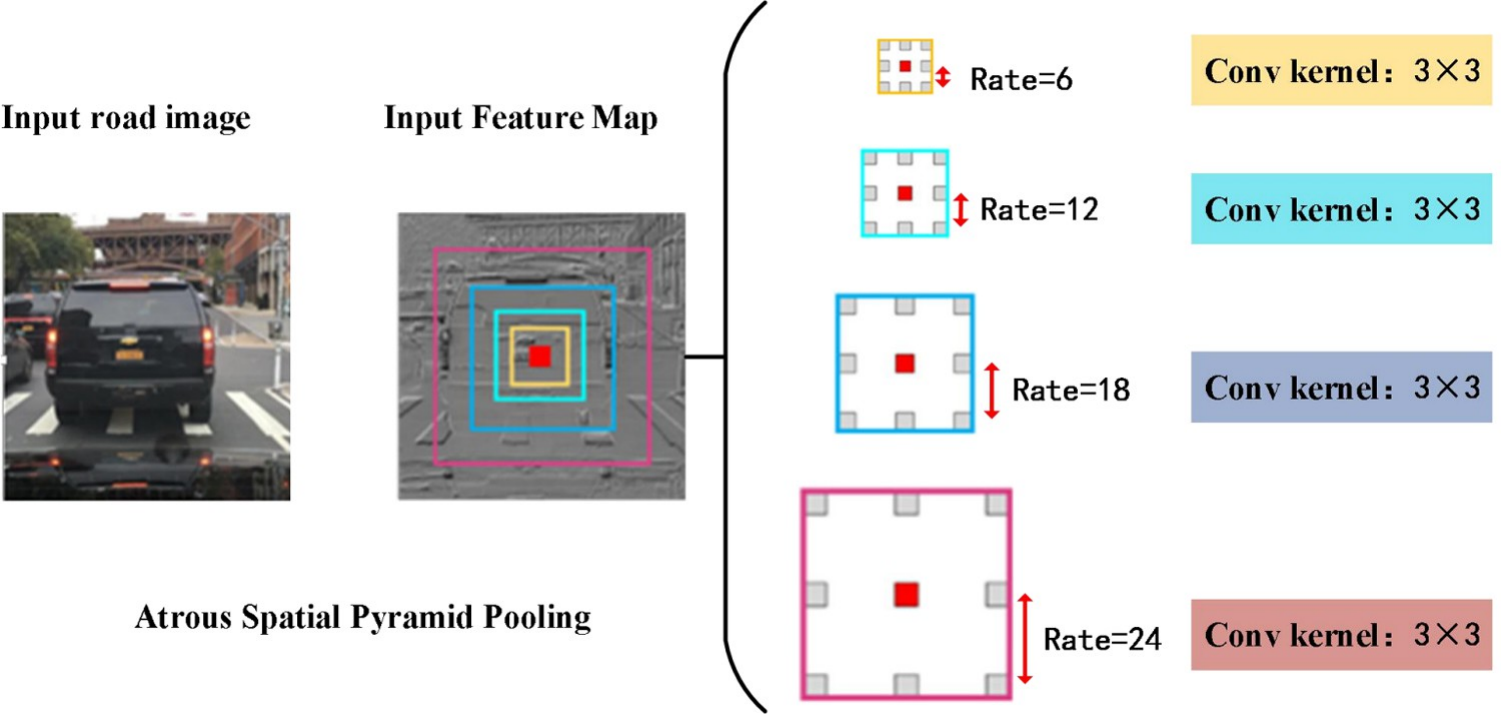
Architecture of ASPP.

ASPP also has the function of capturing multi-scale context information. In the vehicle detection task branch and the lane line detection task branch, because the vehicle and the lane line are multi-scale, which increases the difficulty of the task, it uses multi-scale features and obtains the bottom up and down. Text information is effective to solve the above-mentioned difficulties. Hole convolution can set different hole rates so that one pixel can acquire multiple receptive fields, thereby acquiring information of multiple scales, which is very useful for target detection and semantic segmentation. In order to further accurately perceive targets of different sizes in the process of extracting features, this article uses ASPP as the enhancement module for feature extraction, and its network structure is shown in Fig 2.

In the above figure, ASPP uses hole convolutions with different void ratios of 6, 12, 18 and 24 to convolve the features, so that the extracted feature maps have different receptive fields, and the feature maps of different receptive fields are merged to capture Contextual information of road images at different scales.

#### 3.2.2. Feature fusion module

One of the difficulties in the vehicle detection task branch and the lane line detection task branch is that the sizes of the two detection targets are different, but both of these task branches use high-level feature layers for task operations, and this high-level feature layer is It is obtained through multiple convolutional layers and down-sampling layers, so the size of the feature layer is usually only a few tenths of the original input size. Once the detected vehicle object has a small pixel in the original image, or the end of the lane line In the original image, it is a small target, so the pixel information of the vehicle and lane line of the small target in the high-level feature layer is even less, which greatly increases the difficulty of detection. Therefore, this paper uses the feature pyramid module to improve the detection accuracy of vehicles and lane lines of different scales.

**Fig 3:**
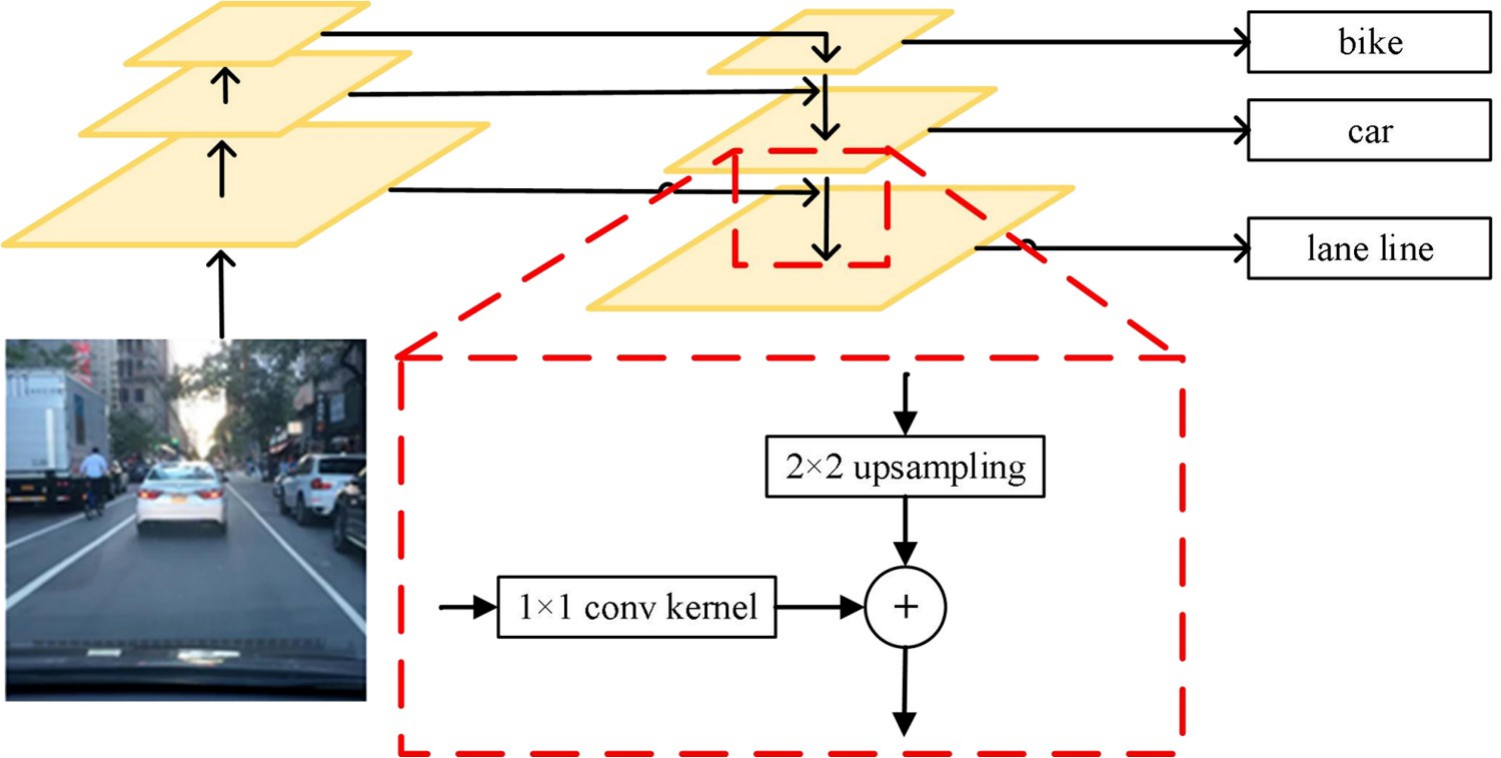
Architecture of pyramid network.

As it is shown in Fig 3, a complete feature pyramid network structure includes two feature map branches, one branch line is bottom-up, this network branch directly uses the feature map extracted by the backbone network, the vehicle detection task branch and the lane line detection task branch Through several stages of convolutional neural networks, the backbone network will extract the feature map group from bottom to top and decreasing in size. The other branch is from top to bottom. By building a small convolutional network, through 2×2 up-sampling operation generates a set of top-down feature maps with an increase in size. The two sets of feature maps are fused with feature maps of the same size, and then the fused feature maps are used for vehicles and Lane line detection. The feature pyramid network can fully mine the spatial semantic information of targets of various sizes and extract features more effectively. At the same time, because the network structure is simple, the increased inference time and additional video memory are less, but it can greatly improve the accuracy of detection and segmentation.

### 3.3. YOLOv4-CBAM block for vehicle detection

**Fig 4:**
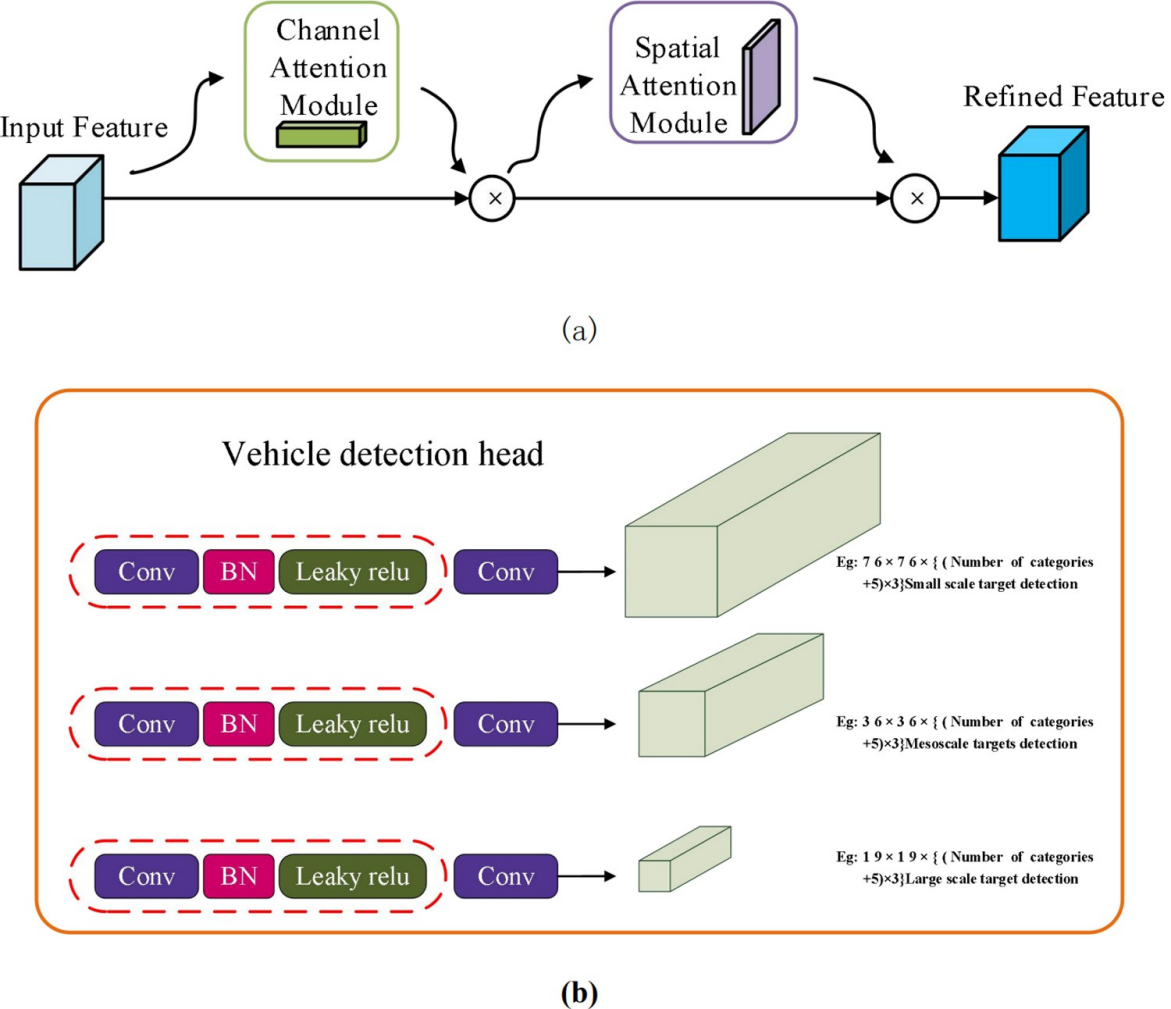
Vehicle detection based on YOLOv4-CBAM. (a) the CBAM block (b) the vehicle detection head block.

Yolov4 (You Only Look Once Version4) is an end-to-end target detection method and has been paid lots attention, because its high real-time and precision. However, the scale of the model and the calculation increase significantly with the increase of the input size, small model and less calculation usually induce lower precision. In order to improve the performance of feature extraction and detection accuracy, the convolutional block attention module (CBAM) is added to YOLOv4 and the vehicle detection block is designed and shown in Fig 4.

As shown in Fig 4, the CBAM block divides the attention process into two independent parts, namely channel attention module and spatial attention module. This can not only reduce parameters and computing power, but also ensure that it can be integrated into the existing network architecture as a plug and play module. The vehicle detection head block mainly has three branches: convolution, batch normalization, and Leaky ReLU activation function. Conv represents 1×1 convolution, and the output of the three branches is The dimensions are 76×76×{(number of categories+5) ×3}, 38×38×{(number of categories+5) ×3}, 19×19×{(number of categories+5) ×3}, {(Number of categories +5) ×3} where 5 represents 4 regression parameters (that is, the position information of the target vehicle in the road image, that is, the x and y coordinate information of the center point of the anchor point box of the vehicle label and the length and width of the anchor point box The value of) plus 1 confidence level, 3 means that each position predicts 3 anchor boxes of different scales, that is, the detection anchor boxes of three different scale vehicles of large, medium and small.

### 3.4. Asymmetric SegNet block for lane detection

**Fig 5:**
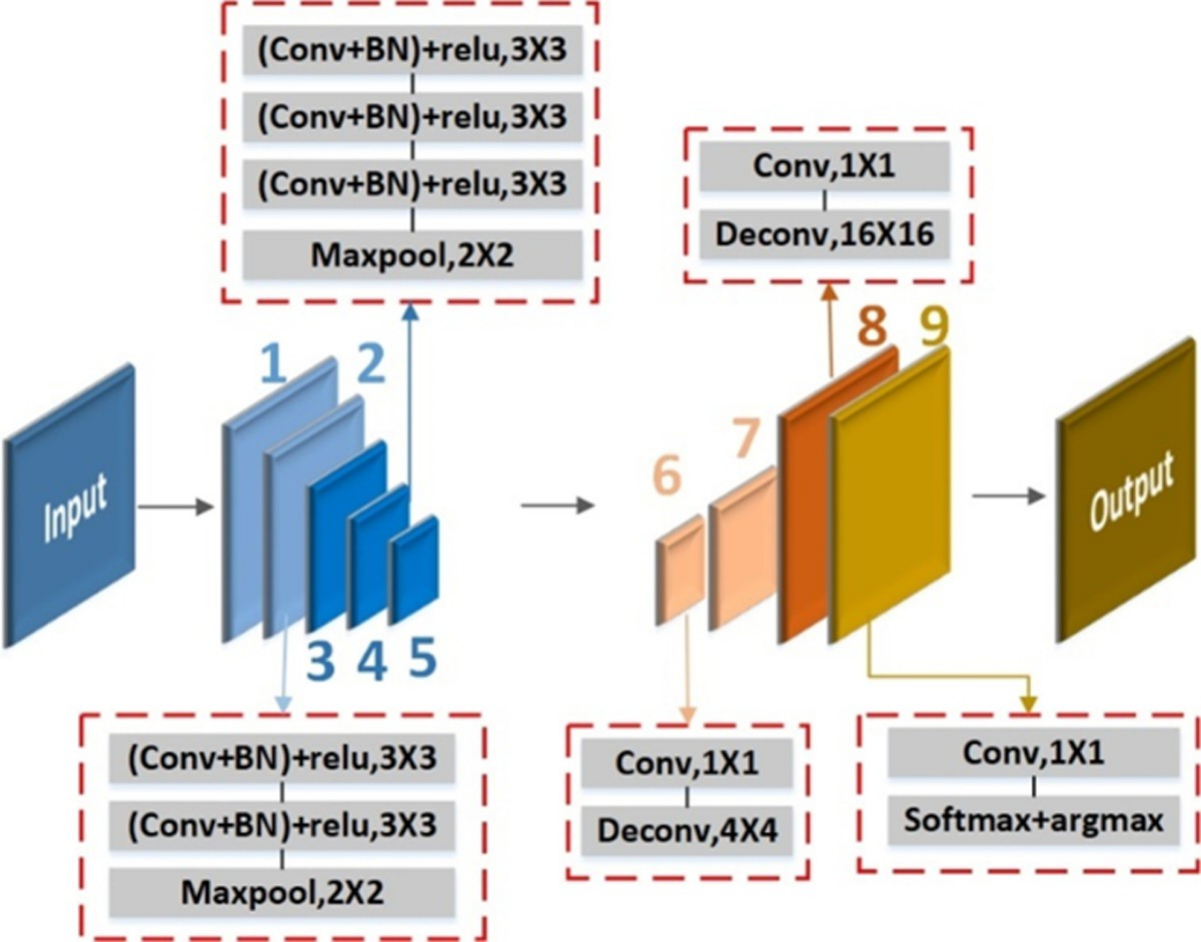
Architecture of improved asymmetric SegNet.

SegNet has a very good segmentation effect in the field of biomedicine and is with excellent performance. Considering that the lane line segmentation is similar to the blood vessel segmentation in the medical field, this paper selects the SegNet lane line detection head module. In order to enhance the real-time performance, the SegNet is changed to an asymmetric network architecture of "more encoding-less decoding", so as to reduce the number of image channels to reduce the hyperparameters of the model. Besides, the 1×1 convolution kernel and batch normalization layers are employed to reduce calculation and accelerate the convergence. The architecture of improved asymmetric SegNet is shown in Fig 5.

**Fig 6:**
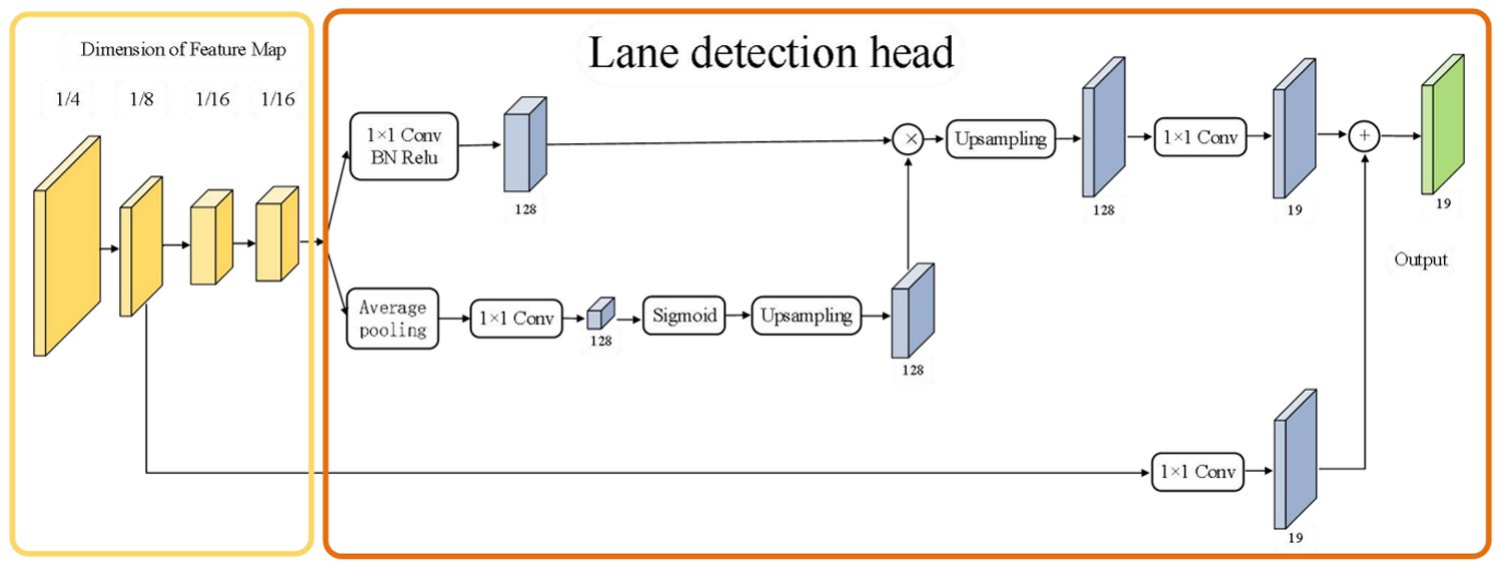
Lane detection block.

According to asymmetric SegNet, the lane detection block is designed and shown in Fig 6. It can be seen that the lane line detection head module of this article performs two 2×2 up-sampling operations on the basis of the maximum feature map output by the FPN, and the feature maps are merged sequentially to obtain features of the same size as the original image, and finally Lane line detection. The first feature fusion of this module is to merge the feature map with the feature map after the standard convolution operation after the average pooling and upsampling of the feature map. The second time is to fuse the FPN output size to 1/8 of the original image input. The feature map is fused with the feature map before output. The two fusions can better extract the semantic information of the lane line, which is beneficial to the detection of the lane line.

### 3.5. Improved focal loss function

The loss function of the vehicle and lane line multi-task joint detection model is more complicated than single vehicle or lane line detection, because it is necessary to measure the loss of vehicle and lane line detection at the same time. Therefore, the loss function of the joint detection model is designed based on focal loss function, and it mainly divided into three parts, including the loss of the regression box, classification and confidence.

The classification function uses Focal Loss [35]. Focal Loss can solve the problem of imbalanced sample proportions. It is based on the cross entropy (CE) of binary classification, and it can be formulated as:

$$FL(p_{t})=-\partial t{\left(1-p_{t}\right)}^{\gamma }\;\log(p_{t})$$ (1)

The loss of the regression box uses the DIoU loss function and it can be expressed as:

$$L_{DIoU}=1-IoU+\frac{\rho^{2}(b,b_{gt})}{c^{2}}$$ (2)

Where *b* is the center point of the detection frame, *b*_*gt*_ is the center point of the target frame, *ρ*(⋅) is the calculated Euclidean distance, and *c* is the diagonal length of the smallest rectangular frame that covers both the detection frame and the target frame.

*L*_*DIoU*_ minimizes the center distance between the detection frame and the target frame by penalizing the distance between the center point of the detection frame and the target frame, and accelerates the convergence speed. In addition, divide by *c* in the denominator term, so that *ρ*^2^(*b*,*b*_*gt*_)/*c*^2^ has nothing to do with the size of the detection frame and the target frame.

Use Dice as the loss function for lane line detection. Dice loss function is a semantic classification loss commonly used in medicine. It has a good segmentation effect on segmenting medical images such as blood vessels. Considering that this paper does two-class semantic segmentation of lane lines, and blood vessel segmentation is very similar, so the Dice loss function is used and formulated as:

$$L_{Dice}=1-\frac{2|\mathrm{Pr}ed+GT|}{\left|\mathrm{Pr}ed|+|GT\right|}$$ (3)

Where the Pr*ed* represents the predicted segmentation image, *GT* represents the real segmentation image, and |⋅| is the pixel summation operation.

Therefore, the total loss function would be expressed as:

$$L_{all}=L_{DIoU}+FL(p_{t})+L_{Dice}$$ (4)

where the *L*_*DIoU*_+*FL*(*p*_*t*_) is the supervision of target detection, and the *L*_*Dice*_ is the supervision of semantic segmentation. There is a certain degree of competition between the two tasks in the optimization direction. If the ratio of the two is selected incorrectly, it is easy to fall into the local maximum. Excellent, how to choose the ratio coefficient of the two is a difficult problem. For this reason, a dynamic adjustment loss function is designed to dynamically adjust the scale coefficients of the two tasks, and it is formulated as:

$$L_{all}=\frac{mAP_{b}}{mAP_{c}+\varepsilon }(L_{DIoU}+FL(p_{t}))+\frac{IoU_{b}}{IoU_{c}+\varepsilon }L_{Dice}$$ (5)

Where the *mAP*_*b*_ is the optimal mAP for single task detection, the *mAP*_*c*_ is mAP of the current model, the *IoU*_*b*_ is the optimal IoU for single-task semantic segmentation, and the *IoU*_*c*_ is the IoU of current model. The value of ε is 10^(-5) and designed to prevent the division from overflowing. In order to regularize the dynamic coefficients *mAP*_*b*_/(*mAP*_*c*_+*ε*), *IoU*_*b*_/(*IoU*_*c*_+*ε*) into a unit vector, and further optimized as:

$$L_{all}=\frac{e^{\frac{mAP_{b}}{mAP_{c}+\varepsilon }}}{e^{\frac{mAP_{b}}{mAP_{c}+\varepsilon }}+e^{\frac{IoU_{b}}{IoU_{c}+\varepsilon }}}\left(L_{DIoU}+FL(p_{t})\right)+\frac{e^{\frac{IoU_{b}}{IoU_{c}+\varepsilon }}}{e^{\frac{mAP_{b}}{mAP_{c}+\varepsilon }}+e^{\frac{IoU_{b}}{IoU_{c}+\varepsilon }}}L_{Dice}$$ (6)

### 3.6. Algorithm

According to the proposed network and improved blocks in each module, the pseudo code of the proposed method is designed and the process of one step-by-step training method is illustrated in Algorithm 1.

**Algorithm 1:** One step-by-step Training Method. First, we only train Encoder and Vehicle Detect head. Then we freeze the Vehicle Detect head as well as train Lane Detect head and Encoder head. Finally, the entire network is trained jointly for all three tasks.

**Input**: Target neural network *F* with parameter group: *θ* = {*θ*_*enc*_,*θ*_*vdet*_,*θ*_*ldet*_};// *enc*:An improved feature extraction module MobileNetV3 shared by segmentation head and detection head. Includes Multi-scale receptive field module and feature fusion module. *vdet*: YOLOv4-CBAM block for vehicle detection. CBAM module is integrated in the convolution layer after ASPP. *ldet*: Asymmetric SegNet block for lane detection. SegNet is changed to an asymmetric network architecture of "more encoding-less decoding".

Training set: *τ*;

Threshold for convergence: *thr*;

Loss function: *L*_*all*_

**Output**: Well-trained network: *F*(x;*θ*)

1: **Procedure** TRAIN(*F*,*τ*)

2: **repeat**

3: Sample a mini-batch (x_*s*_,y_*s*_) from training set *τ*.

4: $l\leftarrow L_{all}(F(x_{s};\theta ),y_{s})$

5: $\theta \leftarrow \arg\min_{\theta }l$

6: **until** l<thr

7: *θ*←*θ*\{*θ*_*ldet*_}//Freeze parameters of Lane Detect head.

8: TRAIN(*F*,*τ*)

9: *θ*←*θ*∪{*θ*_*ldet*_}\{*θ*_*vdet*_}//Freeze parameters of Vehicle Detect head and activate parameters of Lane Detect head.

10: TRAIN(*F*,*τ*)

11: *θ*←*θ*∪{*θ*_*vdet*_}//Activate all parameters of the neural network.

12: TRAIN(*F*,*τ*)

13: return Trained network *F*(x;*θ*)

## 4. Experimental results

### 4.1. Datasets and augmentation

**Fig 7:**
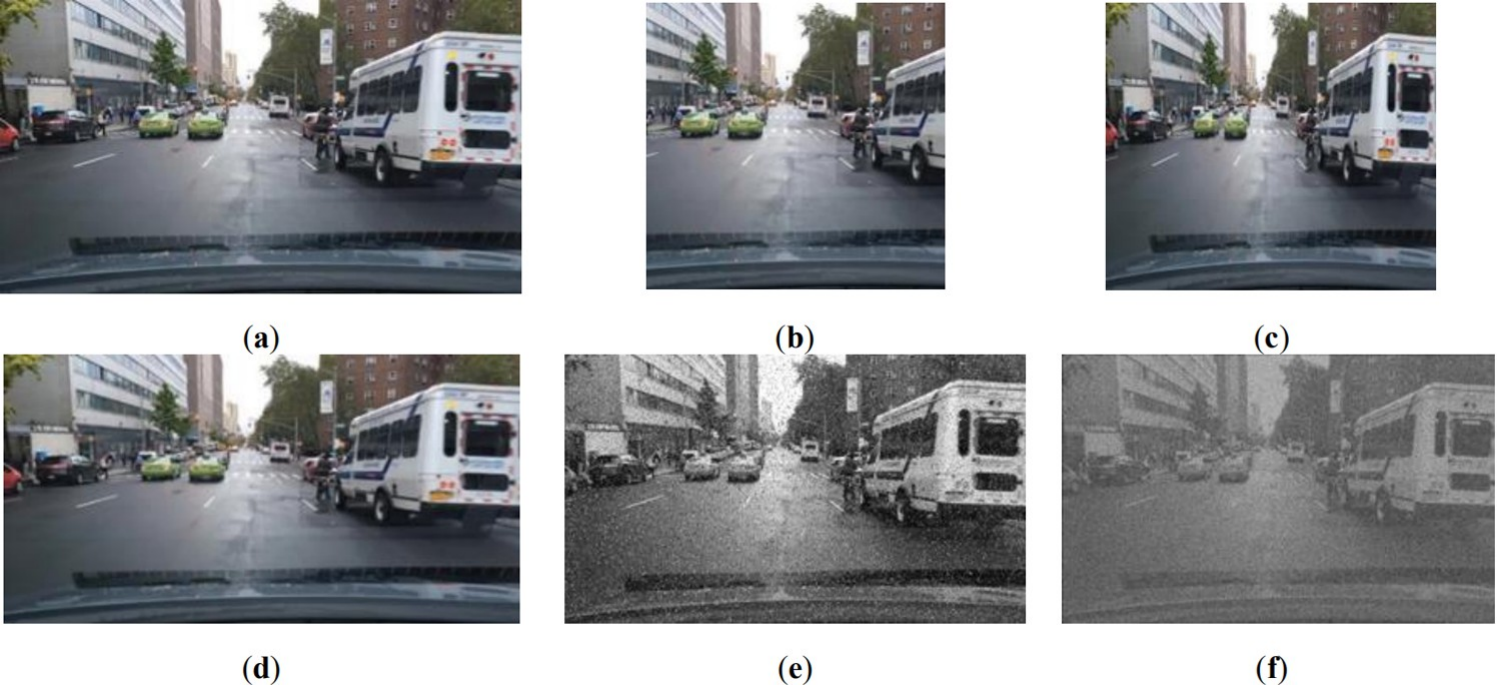
Image augmentation. (a) original image; (b) cropped image; (c) scaled image; (d) flipped image; (e) degraded image with salt noise; (f) degraded image with Gaussian noise.

The car following scene is the driving scene with the largest proportion in the natural scene. The BDD100K data set is employed as the train set, and the car following scene in the BDD100K would attach great attention. The experiment mainly detects the five categories of cars, buses, bicycles, trucks and motorcycles and divides the lane lines. In order to enhance the performance of the algorithm and improve the robustness of the model, some data augmentation methods are applied and the samples are shown in Fig 7.

As it is shown in Fig 7, (a) is an original image in BDD100K, and (b)~(f) are augmented results of original image with the cuts, scales, level flips, salt and pepper noise, Gaussian noise, respectively.

### 4.2. Implementation details of experiment

The experiment was conducted under the environment of Intel(R) Xeon(R) E5-2630, 20-core 40-thread CPU, 128G memory, and 16GB NVIDIA Tesla P100 graphics card. In this paper, the Adam algorithm is used to optimize the model parameters. The weight decay is 5x10^-4^, the initial learning rate is 1x10^-3^, and the learning rate is decayed once in each round. The decay rate is 0.95. A total of 100 rounds are trained. There are 1000 batches in the round, with 70 training samples in each batch.

This paper will use the KITTI data set with both vehicle annotations and lane markings to further verify the model, optimize the performance of the model and complete the comparative evaluation. And we also collected Chongqing traffic road images as a test data set to verify the effect of the model, including two periods of day and night to test the generalization performance of the improved vehicle and lane line joint detection model.

### 4.3. Results and analysis

#### 4.3.1. Loss function analysis

In order to evaluate the effectiveness of the dynamic adjustment loss function, a comparative experiment is conducted. Because the loss value of the YOLOv4-CBAM branch is very large, while the loss value of the asymmetric SegNet branch is very small at first. Therefore, we test the configuration ratio of the loss function with 1:10, 1:50, 1:100, and dynamic adjustment method, and the results is shown in Table 1.

**Table 1:** Comparison of the configuration ratio of the loss function.

Configuration ratio (YOLOv4: SegNet)	Convergence time (h)	IoU	Precision	Recall	AP_50_	F1
1 : 10	76.3	75.5	78.4	61.3	62.5	66.8
1 : 50	68.7	74.9	78.6	61.7	63.2	67.1
1 : 100	72.6	75.1	78.7	61.8	62.9	67.3
Dynamic Adjustment	**60.4**	**75.9**	**79.2**	**62.2**	**63.7**	**67.9**

As it can be seen from Table 1 that the dynamically adjusted loss function can achieve convergence faster. In addition, using the dynamically adjusted loss function for training can improve the detection accuracy and segmentation accuracy of the model. Compared with fixed configuration ratio, dynamic adjustment has a certain degree of improvement in AP50, Recall, F1, Precision, and IoU.

#### 4.3.2. Ablation experiment

In order to better analyze the multi-task joint model algorithm, this paper does a detailed ablation comparison experiment. Table 2 shows the multi-task joint algorithm ablation experiment. Base indicates that MobileNetV3 is used as the backbone network, and the multi-task joint algorithm of multi-scale receptive field module (ASPP) and feature pyramid network (FPN) is not added.

**Table 2:** Ablation study of ASPP and FPN blocks.

Backbone	FPS	Precision	Recall	AP_50_	F1	IoU
Base	**49.3**	73.4	56.9	57.6	63.2	70.0
Base+ASPP	39.8	76.6	59.5	60.2	65.1	73.7
Base+FPN	33.4	77.2	60.3	61.1	65.9	72.9
Base+ASPP+FPN	28.7	**79.2**	**62.2**	**63.7**	**67.9**	**75.9**

As can be seen from Table 2, the introduction of ASPP and FPN in the multi-task joint algorithm has a very good improvement effect. After the introduction of ASPP, it surpasses base network by a large margin of +3.2 Precision, +2.6 Recall, +2.6 AP50, +1.9 F1 and +3.7 IoU, respectively. For FPN module, it could obtain improvement with +3.8 Precision, +3.4 Recall, +3.5 AP50, +2.7 F1 and +2.9 IoU compared base network. Further, the introduction of ASPP and FPN surpasses base network by a large margin of +5.8 Precision, +5.3 Recall, +6.1 AP50, +4.7 F1 and +5.9 IoU, respectively.

**Fig 8:**
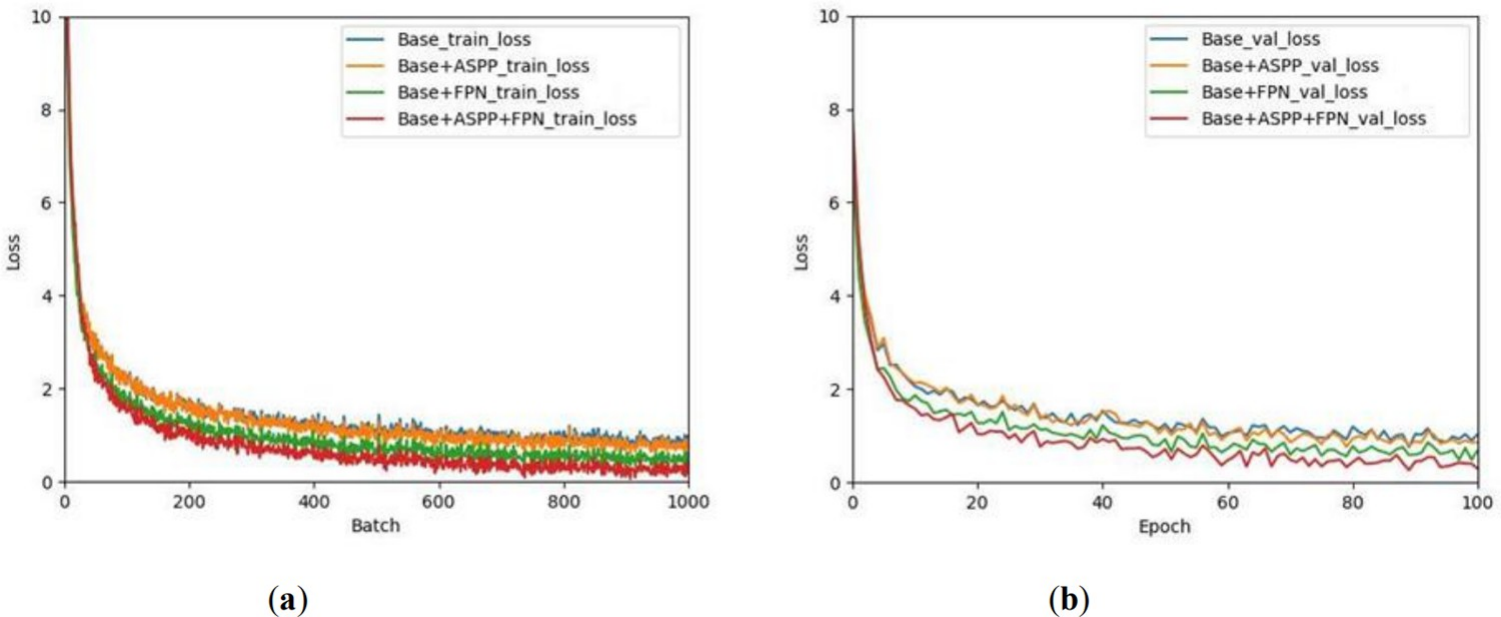
Curves of loss. (a) train loss; (b) total loss.

For the model training process, draw three groups of ablation experimental training and overall loss curve graphs using MobileNetV3 as the backbone network without adding multi-scale receptive field module (ASPP) and feature pyramid network (FPN), as shown in Fig 8.

It can be seen from the Fig 8 that the training loss of the three models drops faster in the initial stage of the network training, and MobileNetV3 is used as the backbone network, and the model loss value of the multi-scale receptive field module and the enhanced feature pyramid network has the fastest decline, and The value has been lower than the loss value of other models, and finally tends to 0. The smaller the loss function value, the better the performance of the model, so the Base+ASPP+FPN model used in this article has better prediction performance.

From the perspective of increased time overhead, the time overhead of introducing FPN is greater, and the time overhead of ASPP is smaller. Although the FPS dropped from 49.3 frames to 28.7 frames after the introduction of ASPP and FPN at the same time, it is still worthwhile to introduce ASPP and FPN after measuring the time cost and accuracy improvement. The experimental results show that the multi-scale receptive field module and the enhanced feature pyramid network play a very good improvement effect, so that the introduction of a lightweight network not only does not cause a decrease in the accuracy of detection and segmentation, but has a certain degree of accuracy improvement.

#### 4.3.3. Comparison of different models

In order to verify the effectiveness of the designed multi-task joint algorithm, we conduct a statistical analysis of the experimental results. The performance of the multi-task joint algorithm and its comparison model experimental data are shown in Table 3 (black font is the best performance).

**Table 3:** Comparison of single model.

Model	FPS	Precision	Recall	AP_50_	F1	IoU
YOLOv4	17.3	78.9	61.3	62.6	67.2	—
SegNet	8.9	—	—	—	—	74.7
Proposed model	**28.7**	**79.2**	**62.2**	**63.7**	**67.9**	**75.9**

According to Table 3, it can be seen that the designed multi-task joint algorithm has reached the best performance in all performance indicators. The experimental results show that the proposed model surpasses the YOLOv4 by a large margin of +1.1 AP50, +0.9 Recall, +0.7 F1 and +0.3 Precision, and surpasses the SegNet by a large margin of +1.2 IoU on BDD100k. At the same time, the detection speed is 1.7 times and 3.2 times of YOLOv4 and SegNet, respectively. It could be found that proposed multi-task joint algorithm trends to obtain higher accuracy although it processes two tasks at the same time. It is worth noting that the detection and segmentation speed of the multi-task joint algorithm is much higher than that of YOLOv4 and SegNet. This is because the multi-task joint algorithm uses MobileNetV3 as the backbone network, which greatly reduces the amount of network parameters.

**Fig 9:**
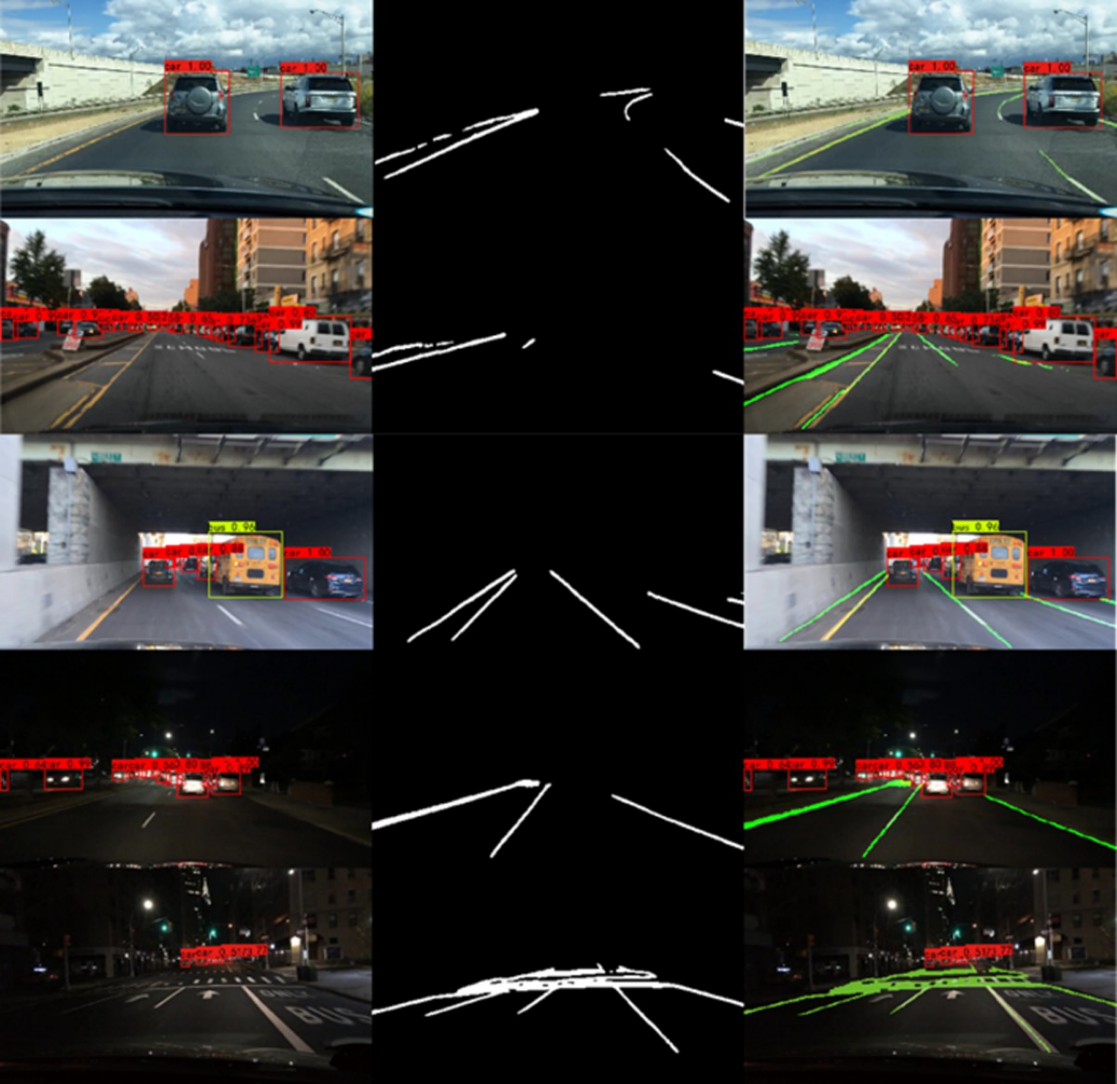
Results of vehicle and lane detection. (a) vehicle detection by YOLOv4-CBAM, (b) lane detection by asymmetric SegNet, (c) vehicle and lane detection by proposed method.

Fig 9 shows the comparison of the vehicle single-task branch and the lane-line single-task branch and the multi-task joint network in the vehicle and lane line joint detection model of the multi-task convolutional neural network. It could be found that the two task branches have completed their task modules well, and they are also well reflected in the overall model, indicating that the improved method is effective.

**Fig 10:**
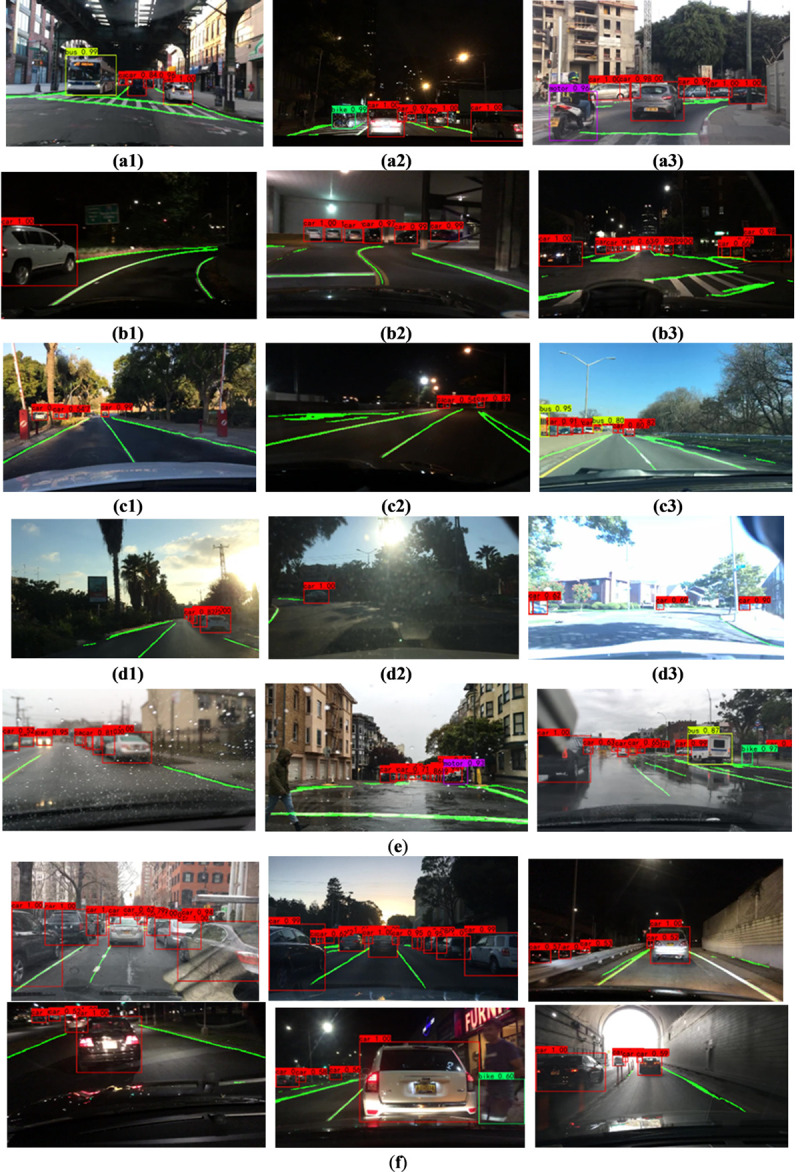
Results of detection under different scenes. (a1) bus scene, (a2) bicycle scene, (a3) motorcycle scene, (b1) curved lane scene, (b2) curve and straight lane scene, (b3) chaotic and complicated lane scene, (c1) small target vehicle in clear day, (c2) small target vehicle at night, (c3) small target vehicles include buses, (d1) sufficient lighting, (d2)strong light, (d3) exposure scene, (e) rain scene, (f) car-follow scene.

Since there are four types of vehicles in this experiment: cars, buses, bicycles, and motorcycles, the weather conditions in the test set are also different. In addition, the lane lines of the experimental subjects can be divided into straight lines and curved lines. The vehicle and lane line joint detection model should also be suitable for common car following scenarios, so as to ensure that the driving assistance system can provide the driver with decision-making information when the vehicle is following the car. The detection results of the vehicle and lane line joint detection model under six different situations are shown in Fig 10.

As it shows in Fig 10, (a1)-(a3) are the detection results of different vehicle types, namely buses, bicycles and motorcycles. (b1)-(b3) are scene detection diagrams where the lane line is a full curve, the lane line curve is added with a straight line, and the lane line is chaotic, indicating that the model can accurately detect no matter what type of lane line appears. (c1) is the detection of vehicles in sunny weather and under the occlusion of a large number of trees. (c2) is the detection of small target vehicle scenes at night. The human eye may not be able to judge the remote vehicle, but this model can accurately detect. (c3) is a small target vehicle that includes a bus, which proves that not only a normal car, but the bus can also detect the location at the far end. (d1)-(d3) are the detection results of vehicles and lane lines under different light conditions. When the light of (d1) is sufficient, both the vehicle and the lane line can be well detected. Although vehicles and lane lines can also be detected in (d2) and (d3), there are cases of missed detection, especially the detection of lane lines. (e) is the detection effect map on rainy days. Rainy days will firstly make the ground wet, and secondly, rain water will accumulate on the windshield in front of the car, increasing the difficulty of lane line and vehicle detection. (f) is the detection effect picture under the car following scene. It can be seen from the figure that proposed model is suitable for car following scenes and can provide good decision-making information and intelligent early warning for the speed of driver when following the car, ensuring the safety of following car driving.

Further, some comparative experiments in BDD100K dataset with the state-of-the-art models are carried out, and the performance of target detection or semantic segmentation for each model is shown in Table 4.

**Table 4:** Comparison with state-of-the-art models.

Model	FPS	Recall	AP_50_	IoU
DLT-Net [36]	9.3	**89.4**	**68.4**	71.3
MultiNet [37]	8.6	81.3	60.2	71.6
Faster R-CNN [38]	5.3	77.2	55.6	—
Proposed model	**28.7**	62.2	63.7	**75.9**

According to Table 4, compared with the state-of-the-art models, the proposed method obtains 63.7% in AP50 and performs second only to DLT-Net. Besides, it could be found that the proposed model holds the best performance in IoU and surpasses the DLT-Net by a large margin of +4.6 IoU though it performs poor in Recall. Moreover, the FPS of the proposed method is 28.7 and achieves the highest real time, which outperforms the other state-of-the-art models by a wide margin.

#### 4.3.4. Comparison of different datasets

After comparing with the single-task model experiment, the comparison of vehicle and lane line by the improved MobileNetV3 joint detection method on different data sets is carried out, and the results are shown in Table 5.

**Table 5:** Comparison of different datasets.

Datasets	AP_50_	F1	Average detection time (ms)
KITTI	63.8	66.3	32.3
BDD100K	63.7	67.9	28.7

It can be seen from Table 4 that the joint detection method of vehicles and lane lines performs well on both types of data sets. The difference between the AP_50_ index and the F1 score value is only 1.6. In terms of the average detection time of a single sheet, the test time on the BDD100K data set is 0.88 times that of the KITTI dataset. This may be because the modified method is trained on the training set of the BDD100K model.

**Fig 11:**
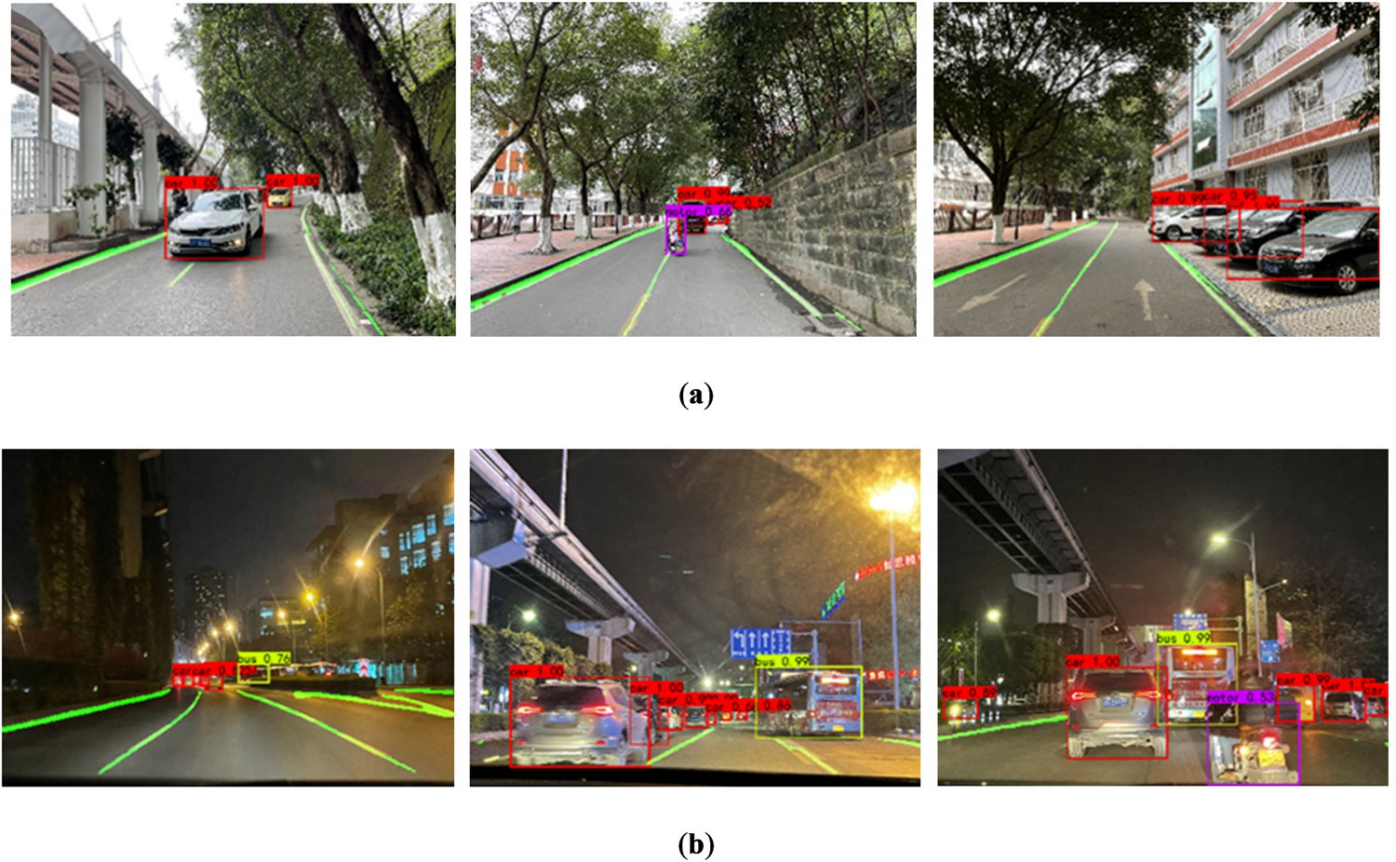
Vehicle and lane detection in Chongqing. (a) sunny road scene, (b) night road scene.

Moreover, the improved vehicle and lane line joint detection algorithm is also used to detect the traffic scene in Chongqing, and the detection effect is shown in Fig 11.

As it shows in Fig 11, the vehicle and lane line can still be detected well in the car following scene. The use of Chongqing road images to test the generalization performance of the model shows that the model can correctly detect vehicles and lane lines at the same time, saving the time of superimposing the two models, and the accuracy rate is also guaranteed.

## 5. Conclusions

This paper improves a vehicle and lane line multitasking joint detection model suitable for car following scenes, which can handle vehicle target detection and lane line two-class semantic segmentation at the same time. In order to reduce the amount of model parameters and improve the speed of detection and segmentation, the lightweight network MobileNetV3 is employed as the backbone network, which greatly improves the detection speed of the network. Meanwhile, in order to improve the detection accuracy and segmentation accuracy of the algorithm, this paper introduces ASPP and FPN in the multi-task network to improve the ability of the network to extract features, thereby improving the detection accuracy and segmentation accuracy of the model. In addition, considering the competitive relationship between tasks during model optimization in a multi-task network, a dynamic adjustment loss module is designed so that the ratio between the target detection loss and the semantic segmentation function can be adjusted during the training process.

In order to verify the effectiveness of the multi-task network designed in this paper, we make a detailed experimental comparison in the public data BDD100k. The experimental results show that the multi-task network model designed is 1.1%, 0.9%, 0.7%, and 0.3% higher than the single-task YOLOv4 in AP_50_, Recall, F1, and Precision respectively, and 1.2% higher than the single-task SegNet in IoU. At the same time, the detection speed is 1.7 times and 3.2 times that of YOLOv4 and SegNet, respectively. Moreover, the following car scenes in the test set are tested in the vehicle and lane line detection model, and the results also prove the effectiveness of the proposed model.

Our work focuses on the vehicle and lane line detection, and a joint network was built to detect vehicle and lane line in car following scene, simultaneously. For autonomous driving car, the pedestrian and drivable area detection are also needed as they are crucial for planning the driving route of the vehicle. Therefore, it is necessary to detect the vehicle, lane line, pedestrian and drivable area in single network. Future research should further improve the accuracy and real-time on multitask simultaneous detection for autonomous driving.

## Supporting information

S1 Data (ZIP)
