# Supplementary figures and images for: Simultaneous vehicle and lane detection via MobileNetV3 in car following scene
Source: PLoS One. 2022 Mar 4;17(3):e0264551. doi: 10.1371/journal.pone.0264551 (PMC8896667; doi:10.1371/journal.pone.0264551)

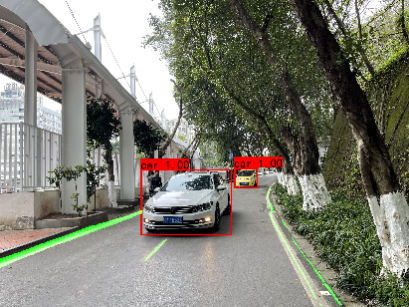

Supplement: S1 Data — (ZIP) [file pone.0264551.s001.zip › support data/1.png]

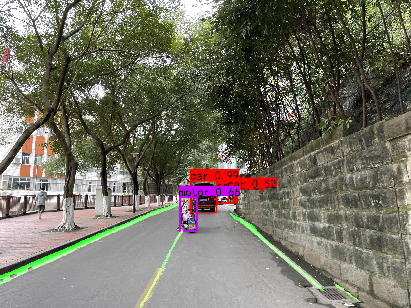

Supplement: S1 Data — (ZIP) [file pone.0264551.s001.zip › support data/2.png]

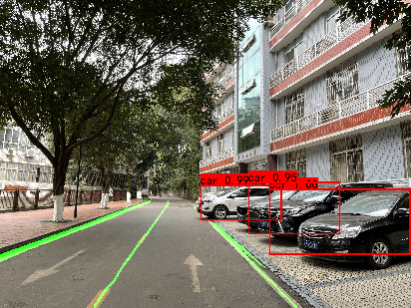

Supplement: S1 Data — (ZIP) [file pone.0264551.s001.zip › support data/3.png]

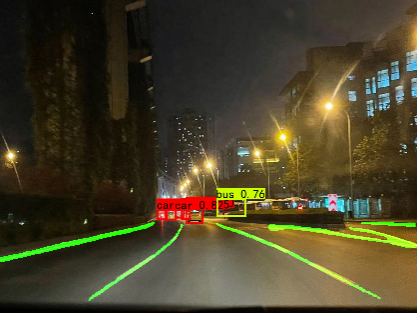

Supplement: S1 Data — (ZIP) [file pone.0264551.s001.zip › support data/4.png]

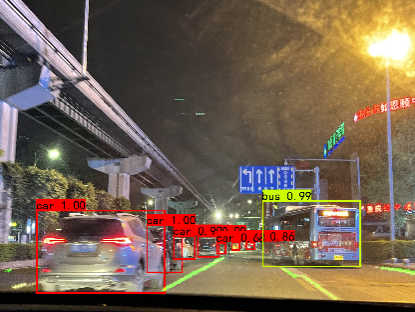

Supplement: S1 Data — (ZIP) [file pone.0264551.s001.zip › support data/5.png]

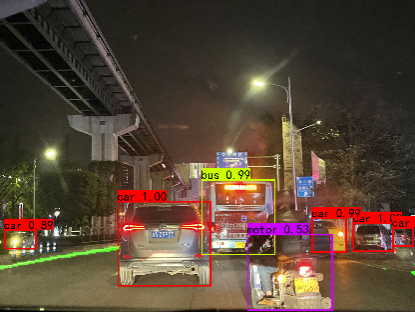

Supplement: S1 Data — (ZIP) [file pone.0264551.s001.zip › support data/6.png]
